# Predictors of Compulsive Sexual Behavior Among Treatment-Seeking Women

**DOI:** 10.1016/j.esxm.2022.100525

**Published:** 2022-05-30

**Authors:** Ewelina Kowalewska, Mateusz Gola, Michał Lew-Starowicz, Shane W. Kraus

**Affiliations:** 1 Department of Psychiatry, Centre of Postgraduate Medical Education, Warsaw, Poland; 2 Institute of Psychology, Polish Academy of Sciences, Warsaw, Poland; 3 Swartz Center for Computational Neuroscience, Institute for Neural Computations, University of California San Diego, San Diego, CA, USA; 4 Department of Psychology, University of Nevada, Las Vegas, NV, USA

**Keywords:** Women, Compulsive Sexual Behavior, Treatment-Seeking, Pornography

## Abstract

**Background:**

Compulsive Sexual Behavior Disorder is currently included in the forthcoming eleventh revision of the International Classification of Diseases (ICD-11); however, prior studies have been conducted mostly on heterosexual, White/European male samples.

**Aim:**

To examine the correlates of compulsive sexual behaviors (CSB) with sociodemographic and sexual history characteristics, as well as predictors of CSB in a sample of treatment-seeking Polish women.

**Methods:**

Six hundred seventy-four (674) Polish women aged 18–66 completed an online-based survey.

**Outcomes:**

Polish adaptation of the *Sexual Addiction Screening Test-Revised* (SAST-PL) was used to assess the severity of CSB symptoms. *Brief Pornography Screen* was used to measure problematic pornography use. The bivariate associations between SAST-PL scores and demographic and sexual history characteristics were also examined. A linear regression analysis was performed to identify variables related to the severity of CSB symptoms.

**Results:**

Thirty one percent (31.8%) of women in the studied sample reported treatment seeking for CSB in the past. Problematic pornography use was the strongest predictor of CSB symptoms. Higher severity of CSB symptoms were observed among divorced/separated and single women compared to those who were married or in informal relation. Severity of CSB was positively related to the number of sexual partners during the last year, number of dyadic sexual intercourse during the last 7 days, and negatively associated with age of first sexual intercourse.

**Clinical Implications:**

Our results suggest that CSB is a significant concern among women and more research is needed to identify protective (eg, relationship status) and risk (eg, problematic pornography use, number of past year sexual partners, frequency of past week masturbation) factors associated with CSB symptom severity among treatment-seeking women.

**Strengths & Limitations:**

Our study is one of very few investigating predictors of CSB among women. Given the lack of precise estimates of the prevalence, as well as lack of psychometrically validated instruments measuring CSB in women, present findings should not be considered indicative of CSB prevalence among Polish women.

**Conclusion:**

The lack of clinical data on women reporting issues with CSB remains an important target for future clinical research exploration.

**Kowalewska E, Gola M, Lew-Starowicz M, et al. Predictors of Compulsive Sexual Behavior Among Treatment-Seeking Women. Sex Med 2022;10:100525.**

## Introduction

Recently, scientists and clinicians have expressed concerns about the lack of gender representation in studies examining the etiology of problematic sexual behavior.^1^ In the past 20 years, a wide body of literature has evolved proposing theoretical approaches such as compulsive sexual behavior,^2^, ^3^, ^4^, ^5^ hypersexuality,^6^, ^7^, ^8^ out-of-control sexual behavior,^9^ sexual addiction or sexual dependence,^10^, ^11^, ^12^ and sexual impulsivity.^13^, ^14^, ^15^ Across the hundreds of studies published in the past 20 years examining problematic sexual behaviors in different populations, most recruited samples have consisted of mostly White/European, heterosexual men.^1^

In 2019, Compulsive Sexual Behavior Disorder (CSBD) was officially included in the forthcoming 11th edition of the International Classification of Diseases (ICD-11; 6C72), and according to World Health Organization's^16^ definition is characterized by a persistent pattern of failure to control intense, sexual impulses or urges, resulting in repetitive sexual behavior over an extended period (eg, 6 months or more) that induces marked distress or impairment in personal, family, social, educational, occupational, or other important areas of functioning.^17^ The decision of WHO is undoubtedly a huge step in understanding CSBD as a distinct disorder,^1^ though questions remain about CSBD classification as an impulse control disorder given preliminary data highlighting similarities of neuronal mechanisms of CSBD to other addictions,^3^^,^^5^^,^^18^ proposed conceptualizations,^17^^,^^19^^,^^20^ and potential therapeutic interventions.^21^, ^22^, ^23^, ^24^

While analyzing the results of scientific investigation to date, Kowalewska and colleagues^25^ noted that most studies (over 99%) examining CSBD in clinical and community were comprised of heterosexual men. After reviewing 58 studies comprising of women, results suggested that compulsive sexual behavior (CSB) symptom severity is generally lower in women relative to men. Furthermore, women reported consuming pornography less often than men and exhibit lower rates of feeling urges to these materials. CSB symptoms (including problematic pornography use) were also found to be positively related to trait psychopathy, impulsivity, sensation seeking, attention-deficit/hyperactivity disorder symptoms, obsessive-compulsive disorder, pathological buying, sexual dysfunctions, general psychopathology, child sexual abuse, while negatively related to dispositional mindfulness.^25^

Given the gender gaps that exists in understanding the etiology of problematic sexual behaviors (including CSBD) in women, the current study seeks to remedy these issues by broadly examining the correlates of CSB with sociodemographic and sexual history characteristics in a sample of treatment-seeking Polish women. Specifically, because we employed a self-report questionnaire that is not based on the CSBD diagnostic criteria proposed by WHO in 2019, we have, therefore, tried to examine the predictors of more broad ‘compulsive sexual behaviors (CSB)’ among women.

At the recruitment stage, we did not screen for compliance with the ICD-11 criteria, we used a questionnaire to measure CSB symptoms severity. We recognize the limitation of using a self-report measure attributed to assessing sexual addiction symptomology^10^, ^11^, ^12^ but believe present results can be used to identify characteristics attributed to CSB symptoms. Therefore, we will use the term CSB instead of CSBD in this article, although we do not know how many women met the ICD-11 criteria. Given the exploratory nature of this study, analyses were conducted to generate hypotheses for future research studies.

## Methodology

### Participants and procedure

Six hundred seventy-four (*n* = 674) White, Polish women aged 18–66 (*M_age_*=29.36; *SD_age_*=8.13) were recruited through an online-based survey gathering knowledge about the frequency of various forms of CSB among women and their broader clinical picture. The survey was also an invitation to participate in the longitudinal project aimed at examining whether the psychological training leads to reduction of CSB symptoms. Upon entering, respondents were informed about the purpose of the study and provided informed consent electronically. Inclusion criteria were being female, aged 18 or older, being sexually active during the last year (including dyadic sexual activity as well as solitary practices – ie, masturbation), and experiencing difficulties with CSB on a subjective level and looking for treatment due these problems Data was gathered from July 2019 to January 2020. Out of 1241 women who opened the survey, 936 filled it in partially, and 674 completed the entire survey providing data sufficient for the analysis.

### Measures

#### Demographics

Participants’ demographic information such as age, marital status, education level and occupation were obtained.

#### Sexual activity

Participants were asked to provide information on sexual activity defined as any sexual activity – solitary (eg, masturbation, pornography consumption) or dyadic (eg, partnered sex, sexual stimulation including foreplay/fondling, oral sex, vaginal, or anal penetrative intercourse) that induces sexual arousal. Specifically, the content of the questions concerned: onset of first sexual intercourse, number of past year sexual partners, onset (ie, age) of pornography viewing, and number of dyadic sexual intercourse, pornography viewing, and frequency of masturbation in the past 7 days.

#### Prior help-seeking for CSB

We assessed women's help-seeking for CSB experience by asking them to indicate ‘Yes’ or ‘No’ to the following question: ‘Have you ever sought professional help for your compulsive sexual behavior?’.

#### Polish version of Sexual Addiction Screening Test-Revised (SAST-PL)

SAST-PL^26^ is a psychometrically validated instrument that measures CSB based on the concept of sex addiction.^10^ The 20-item questionnaire is comprised of 5 subscales: Affect Disturbance, Relationship Disturbance, Preoccupation, Loss of Control, Associated Features. Respondents are asked to answer each item by responding ‘Yes’ or ‘No.’ Higher scores are related to higher CSB symptoms severity. SAST-PL is characterized by a high reliability (*α* =0.90).

#### Brief Pornography Screen (BPS)

The BPS is a 5-item screening instrument that measures problematic pornography use (PPU).^27^ Respondents rate to each statement by answering the question of how often in the last 6 months they took place on a 3-point scale (0 = Never; 1 = Sometimes; 3 = Very Often). The BPS was initially validated on five independent studies on the American and Polish adults (*α* range from 0.90 to 0.92). Scores on BPS range from 0 to 10 with a cut-off value of 4 indicative of possible PPU.

### Statistical analyses

First, we used Pearson Product correlations, Welch *t*-tests and one-way ANOVAs to examine associations between SAST-PL total score and demographics and sexual characteristics. Next, we conducted a linear regression analysis to identify variables related to severity of CSB symptoms (assessed by SAST-PL). All analyses were performed using SPSS-23 (IBM SPSS Statistics for Windows, Version 23.0).

### Ethics

All procedures in this study were carried out in accordance with the Declaration of Helsinki. Research Ethics Committee of SWPS University in Warsaw approved the study. All participants were informed about the scope of the study, and all provided informed and voluntary consent electronically.

## Results

Out of the 674 women, 57.4% (*n* = 387) scored 6 points or higher on the SAST-PL,^26^ indicative of CSB, and 73.3% (*n* = 494) of the sample scored 4 points or higher on the BPS measuring symptoms of problematic pornography use.^27^

Table 1 shows the bivariate associations between SAST-PL total score and sociodemographic and sexual history characteristics. Specifically, we found positive correlations between the SAST-PL total score and BPS total score (*r* = 0.59, *P* < .001), number of past year sexual partners (*r* = 0.34, *P* < .001), and number of past week (7 days) dyadic sexual intercourse (*r* = 0.15, *P* < .01). Negative correlations occurred between SAST-PL total score and age of participants (*r* = −0.08, *P* < .05), onset of first sexual intercourse (*r* = −0.24, *P* < .001), and onset of first pornography exposure (*r* = −0.23, *P* < .001]. Furthermore, women who were during divorce, separation or single scored significantly higher on SAST-PL (*M* = 7.67, *SD* = 4.79) compared to those who were married or in informal relation (*M* = 6.48, *SD* = 4.37), [*t*(672) = 3.26, *P* < .001, Cohen's *d* = 0.26].

**Table 1:** Demographic and sexual history factors associated with women's SAST-R score (n = 674)

		SAST-R score
Study characteristics	%/*M* (*SD*)	*r* or *t*/*F*
Sexual Addiction Screening Test – Revised (SAST-R)† Does not meet cut-off Meet cut-off	6.91 (4.55) 42.6% 57.4%	**-**
Brief Pornography Screen (BPS)† Does not meet cut-off Meet cut-off	2.75 (2.96) 26.7% 73.3%	***r* = 0.59**⁎⁎⁎
Age	29.36 (8.13)	*r* = −0.08*
Relationship status Married or informal relation During divorce, separation, or single	64.1% 35.9%	***t* = 3.26**⁎⁎⁎ (Cohen's *d* = 0.26)
Education level High school or less College (still at school) Graduate or postgraduate degree	25.7% 18.5% 53.0%	***F* = 6.82**⁎⁎⁎ (Cohen's *f* = 0.13)
Occupation Full time or part time Student/Unemployed	73.0% 27.0%	*t* = −0.90
Prior help-seeking due to CSB Yes No	31.8% 68.2%	***t* = −5.38**⁎⁎⁎ (Cohen's *d* = 0.45)
Onset of first sexual intercourse	N = 652 17.83 (3.02)	***r* = −0.24**⁎⁎⁎
Number of sexual partners during the last year	N = 558 3.28 (5.45)	***r* = 0.34**⁎⁎⁎
Number of dyadic sexual intercourse during the last week (7 days)	N = 430 3.21 (3.45)	***r* = 0.15**⁎⁎
Onset of first pornography exposure	N = 649 12.75 (4.37)	***r* = −0.23**⁎⁎⁎
Time spent on pornography during the last week (7 days) None 59 minutes or less 60–119 minutes 120 minutes and more	50.0% 24.0% 11.6% 14.1%	***F* = 33.69**⁎⁎⁎ (Cohen's *f* = 0.38)
Number of masturbations during the last week (7 days)	N = 516 3.89 (3.82)	***r* = 0.35**⁎⁎⁎

⁎ *P* < .05.

⁎⁎ *P* < .01.

⁎⁎⁎ *P* < .001. Note. Bolded items remained statistically significant after adjusting for Type 1 error.

† Cut-off scores based on research including male participants.

Another significant difference occurred in case of education level, with women reporting high school or less education obtained the highest SAST-PL total score (*M* = 7.60, *SD* = 4.41), followed by women in college scored slightly lower (*M* = 7.54, *SD* = 4.37), and lastly, women with graduate or postgraduate degree having the lowest SAST-PL total score (*M* = 6.27, *SD* = 4.59), [*F*(2,652) = 6.82, *P* = .001, Cohen's *f* = 0.13]. As it turned out, women who sought past help for CSB obtained significantly higher scores on the SAST-PL (*M* = 8.26, *SD* = 5.04) compared to women who had not sought out help in the past (*M* = 6.28, *SD* = 4.17), [*t*(672) = −5.38, *P* < .001, Cohen's *d* = 0.45]. Finally, the more time women spent on pornography during the last week (7 days), the higher score they obtained in SAST-PL [*F*(3,668) = 33.69, *P* < .001, Cohen's *f* = 0.38]. Specifically, women who were not watching pornography in the last week obtained a mean score of 5.59 (*SD*=4.21), followed by those who watched pornography for 59 minutes or less – 6.93 (*SD* = 4.27), women who spent 60–119 minutes on pornography – 8.26 (*SD* = 4.07), and lastly, women who devoted 120 minutes or more to pornography consumption – 10.32 (*SD* = 4.51). We did not find an association between SAST-PL total score and occupational status.

Lastly, a simple linear regression was conducted to identify predictors of CSB as assessed by SAST-PL (as a continuous score) in a sample of treatment-seeking Polish women. To reduce effects of Type I error, only variables significant at *P* < .01 were entered into the model (see Table 1). Because prior help-seeking for CSB was highly correlated with CSB, and to minimize the possible effects of multicollinearity, we decided to not include this variable into the regression analysis. The model was significant, *F*(9, 273) = 31.792, *P* < .001, *R^2^* of 0.512. In particular, we found that the BPS total score was the strongest predictor of the CSB (SAST-PL scores) in women (*β* = 0.83, *P* < .001). Furthermore, we found that onset of first sexual intercourse (*β* = −0.21, *P* < .01), number of past year sexual partners (*β* = 0.23, *P* < .001), number of past week masturbations (*β* = 0.22, *P* < .001), and relationship status (*β* = −0.92, *P* < .05) were also significant predictors of CSB (SAST-PL) scores among this sample of help-seeking women (see Table 2).

**Table 2:** Statistical predictors of compulsive sexual behavior (CSB) measured by SAST-R among women

Study characteristics	B	SE B	t	95% CI
(Constant)	8.25	1.35	6.13	[5.60, 10.90]⁎⁎⁎
Relationship status	**−0.92**	0.47	**−**1.95	[**−**1.85, 0.01]*
Education	**−**0.08	0.24	**−**0.33	[**−**0.54, 0.38]
Onset of first sexual intercourse	**−0.21**	0.07	**−**3.13	[**−**0.34, **−**0.08]⁎⁎
Number of sexual partners during the last year	**0.23**	0.04	5.84	[0.15, 0.30]⁎⁎⁎
Number of dyadic sexual intercourse during the last 7 days	0.04	0.06	0.59	[**−**0.09, 0.16]
Onset of first pornography exposure	**−**0.02	0.05	**−**0.31	[**−**0.11, 0.08]
Time spent on pornography during the last 7 days	**−**0.28	0.21	**−**1.34	[**−**0.70, 0.13]
Number of masturbations during the last 7 days	**0.22**	0.06	3.51	[0.10, 0.34]⁎⁎⁎
Brief Pornography Screen (BPS)	**0.83**	0.08	10.27	[0.67, 0.99]⁎⁎⁎

⁎ *P* < .05.

⁎⁎ *P* < .01

⁎⁎⁎ *P* < .001. Relationship status: 0 = divorced/separated/single, 1 = married/partnered; Time spent on pornography during the last 7 days: 0 = none, 1 = 59 minutes or less, 2 = 60–119 minutes, 3 = 120 minutes and more. Note. Linear regression predicting likelihood of the occurrence of CSB symptoms among women. Model summary: *F*(9, 273) = 31.792, *P* < .001 with an R^2^ of 0.512.

## Discussion

Using the Polish adaptation of the *Sexual Addiction Screening Test-Revised* (SAST-PL),^26^ we sought to examine correlates and predictors of CSB symptoms among a sample of treatment-seeking Polish women. Although there are limitations with using this approach, currently there are no psychometrically validated tools validated for assessing CSB (or CSBD) in Polish women. Currently, the lack of clinical data on women reporting issues with CSB remains an important target for future research, particularly since current conceptualizations of the etiology of problematic sexual behaviors are derived from mostly White/European, heterosexual male samples.

Overall, we found that a group of women who had not sought treatment for CSB in the past (68.2% of the whole sample) obtained a mean SAST-PL score that exceed a cut-off value proposed by Carnes.^10^ This finding is in line with an analysis by Kraus and colleagues^29^ showing that 29% of men in their sample who met or exceed the Hypersexual Behavior Inventory (HBI)^30^ total clinical cutoff score, suggesting the presence of a possible Hypersexual Disorder (HD),^6^ were disinterested in seeking treatment for pornography use. However, preliminary data suggests that the probability of seeking treatment for PPU in women is 7 times lower than in men,^31^ though factors that may uniquely contribute to this possible difference have yet to be explored. Given that many women in the study were disinterested in seeking treatment in the past, and nearly 32% of the sample were interested in such treatment, further work is needed to identify current barriers to help seeking for Polish women. Possible explanations could include cultural norms, established gender and social roles for women, religious greater acceptance of men reporting loss of control over sexual behavior, and perceived shame and stigma for women reporting issues with CSB. Dhuffar and Griffits^32^ distinguished 4 main types of possible barriers for women not seeking treatment for sexual addiction (eg, individual, social, research, and treatment); however, future research is needed to identify factors (eg, age, marital status, race/ethnicity, religious beliefs, access to healthcare, co-occurring mental health issues) that prevent women from seeking treatment for CSB.

While examining which of the variables may be predictors of CSB symptoms among women from the studied sample, we showed that in case of women, the strongest predictor of CSB symptoms was the BPS total score. The results obtained in this study also indicated that the following characteristics may be related to the CSB symptoms: onset of first sexual intercourse, number of sexual partners during the last year, number of masturbations during the last week, and relationship status. Due to the lack of similar analysis conducted on women so far, we do not have a reference point for our results. To our knowledge, our study is the first to identify predictors of CSB among Polish women. Our results are similar to a study of a 2017 study on Polish women seeking treatment for PPU^31^ in which they also found a significant relationship between seeking treatment, CSB symptoms (assessed by SAST-PL) and PPU symptom severity (assessed by BPS). Interestingly, we found that it was not the amount of time spent consuming pornography during the past 7 days, but the BPS total score that served as a robust predictor of CSB in women. A possible explanation of this result is the fact that BPS does not focus on the quantitative measure of pornography (ie, quantity and frequency), but instead measures self-perceived consequences attributed to one's pornography consumption. Another noticeable similarity was noted between our investigation and the study of Klein and colleagues^28^ in which the analyses have shown number of sexual partners and high masturbation frequency as predictors of hypersexuality (assessed by HBI)^30^ in women. Research also indicates child childhood abuse, current depression, and substance abuse as predictors of sex addiction,^33^^,^^34^ as well as engagement in religious practices as a predictor of PPU.^31^ These factors which remain salient to CSB in women, were not, however, assessed in the current study and require further consideration in research studies.

Furthermore, we found some significant correlates of CSB symptoms in terms of sociodemographic and sexual history characteristics. For example, higher CSB symptoms severity (SAST-PL total scores) were observed in women who were divorced, separated or single, compare to women who were married or in informal relation. Moreover, SAST-PL total scores were positively related to the number of sexual partners during the last year, number of dyadic sexual intercourse during the last 7 days, while negatively associated with the age of first sexual intercourse. Considering the above result and the fact that Klein and colleagues’^28^ investigation pointed the number of sexual partners as one of predictors of hypersexual behavior, further research is needed to examine dyadic sexual activity among women reporting issues with CSB, as this could reflect an important aspect of the condition that remains understudied in women.

We also explored aspects of pornography consumption and masturbation among our sample of women. As it turned out, the average SAST-PL total score increased with the amount of time devoted to pornography consumption during the past 7 days. CSB symptoms were positively related to BPS scores, number of masturbations during the past 7 days, and negatively associated with the onset of first pornography exposure.

## Limitations

Several limitations of the current study must be considered. First, there is currently no precise estimates of the prevalence of CSBD among women and the current study should not be considered indicative of prevalence of CSBD or CSB among Polish women. Given the lack of instruments measuring CSBD that have been psychometrically validated in samples of women, we do not know, whether the scale we included in our study increased risk of false positives given lack of data assessing factors such as sensitivity and specificity. Second, the survey was advertised using the snowball method among people interested in this subject, so the large number of women declaring prior help with CSB may be due to the group interested in participating in the study itself. Third, our study did not include any measures assessing psychopathology or social desirability/impression management, nor were women interviewed in person by a trained mental health provider. The reliance on self-report data to describe women's experience with CSB should be taken into consideration when interpreting current study findings.

## Conclusions

In summary, present results suggest there is greater need for further exploration of CSB among women, particularly regarding the role of pornography consumption and sexual relationship patterns in the development and maintenance of CSB. Additional studies are needed to determine the prevalence of CSB among women using validated measures which reflect CSBD criteria in ICD-11. Further, research is also needed to examine its co-occurrence with personality, sexual functioning, gambling disorder, substance use, and/or other mental disorders; such data could be used to verify the similarities and/or differences in neuronal mechanisms underlying CSB in women and men.^35^ Lastly, the diagnostic accuracy of commonly used psychometric instruments used to measure symptoms of CSB also requires further exploration, particularly among clinical populations of women which are extremely understudied in low and high income countries.^25^

## Ethics

All procedures in this study were carried out in accordance with the Declaration of Helsinki. Research Ethics Committee of SWPS University in Warsaw approved the study. All participants were informed about the scope of the study and all provided informed and voluntary consent.

## Statement of Authorship

EK contributed to study and methods design, subject recruitment, data collection, data analysis and interpretation, manuscript writing, and obtaining funding. MG contributed to study and methods design, and manuscript writing. MLS contributed to manuscript writing. SWK contributed to data analysis and interpretation, and manuscript writing. All authors provided input, read, reviewed, and approved the final draft of the manuscript.
